# The role of developmental caregiving programming in modulating our affiliation tendency and the vulnerability to social anxiety and eating disorders

**DOI:** 10.3389/fpsyg.2023.1259415

**Published:** 2024-01-04

**Authors:** Marcantonio Gagliardi

**Affiliations:** Institute for Lifecourse Development (ILD), University of Greenwich, London, United Kingdom

**Keywords:** caregiving programming, imprinting, sensitive period, somaticity, social anxiety, eating disorders, evolution, mental disorders

## Abstract

Attachment is the evolutionarily-established process through which humans create bonds with others to receive care from them. The phenomenon is as essential to our physical survival as it is to our psychological development. An increasing number of studies demonstrates that in sensitive periods during the early years of life, our brain circuitry is programmed in the interactions with our caregivers, with the imprinting of information over multiple attachment dimensions. Adopting a basic *brain-computer analogy*, we can think of this knowledge as the psycho-social *firmware* of our mind. According to a recently proposed extension of the classical three-dimensional view, one attachment dimension – *somaticity* – concerns the caregiver’s task of reflecting and confirming the child’s (internal) states – such as sensations, emotions, and representations – to support the child’s ability to identify and define those entities autonomously. Relying on multidisciplinary evidence – from neuroscientific, developmental, evolutionary, and clinical sources – we suggest that somaticity (H1) has the adaptive function to modulate our tendency to comply and affiliate with a reference group but also (H2) increases the vulnerability to developing *Social Anxiety* (SA) and *Eating Disorders* (EDs). We evaluate H1-H2, (1) indicating the evolutionary role of somaticity in modulating our affiliation tendency to optimize the ancestral threat-opportunity balance coming from infectious diseases and (2) showing the deep connection between SA-EDs and the features most closely related to somaticity – interoception and parenting style. Finally, we discuss three relevant implications of H1-H2: (A) Bringing into research focus the adaptive role of our *firmware* knowledge system versus the *hardware* (neural substrate) and *software* (higher cognition) ones. (B) Complementing the well-grounded *Objectification* and *Allocentric Lock Theories*, allowing us to integrate multiple levels of explanation on the etiology of psychopathology. (C) Suggesting the design of new psychological treatments. While not aiming to prove H1-H2, our analysis supports them and encourages their direct testing.

## 1 Introduction

Attachment theory concerns the deep bonds humans create with others to receive care from them (Bowlby, 1969/1982). Such bonds can be formed at any age but are especially evident in infancy and childhood. In fact, we evolved as an altricial species and need to be taken care of by adults for years after birth before becoming independent. During this time, our caregivers are essential to our physical survival and psychological development. Despite not being usually explicitly covered by evolutionary and developmental theories, a growing body of research shows that children are evolutionarily prepared for ***caregiving programming*** – i.e., to be programmed by their caregivers’ operation – over several dimensions (*cf.* 2.2). The particular mechanism – *imprinting* – through which attachment dimensions are acquired consolidates their foundational role and suggests thinking of them as the psycho-social ***firmware*** of our mind. Different fundamental caregiving activities were proposed to induce the acquisition of specific attachment dimensions. For instance, the degrees of the caregiver’s sensitivity and responsiveness provide critical information on future emotional and physical care, corresponding to the child’s *avoidant* and *ambivalent* attachment dimensions (Fraley and Spieker, 2003). Similarly, the caregiving task of reflecting and confirming the child’s (internal) states – such as sensations, emotions, and representations – was suggested to induce the acquisition of the child’s ***somaticity***, the *somatic* attachment dimension (Gagliardi, 2021). In this case, not confirming the child’s states prevents the child from developing the ability to recognize and define their own states, thereby making them dependent on external input to define themselves.

Relying on multidisciplinary evidence – from neuroscientific, developmental, evolutionary, and clinical sources – we hypothesize here that somaticity **(H1)** has the adaptive function to modulate our tendency to comply and affiliate with a reference group but also **(H2)** increases the vulnerability to developing Social Anxiety (SA) and Eating Disorders (EDs). To lay out and substantiate these hypotheses, we proceed through the following sections:

- **Background**. We outline two premises (P1, P2) to H1-H2. P1 concerns the development of human self-awareness and its relation to somatic experience. P2 covers early caregiving programming.
- **Hypotheses**. We show how P1 and P2 lead to H1-H2 and present our hypotheses: (H1) Somaticity has the adaptive value to modulate affiliation but also (H2) the possible drawback of increasing the vulnerability to SA and EDs.
- **Evaluation of the hypotheses**. We evaluate H1-H2. First, we frame the general evolutionary-motivational properties of each attachment dimension. Then, we discuss the adaptive value of somaticity, providing evidence of its role in modulating our tendency to group affiliation depending on the ancestral threat of infectious diseases (H1) and show the close relationship between SA-EDs and the foundational elements of somaticity – interoception and parenting style (H2).
- **Implications of the hypotheses**. Finally, we illustrate the significant consequences of H1-H2. (A) We discuss how our hypotheses support the evolutionary relevance of developmental caregiving programming to mental functioning and its disorders. (B) We show they are consistent with and complementary to the well-grounded *Objectification Theory* (OT) (Fredrickson and Roberts, 1997) and *Allocentric Lock Theory* (ALT) (Riva, 2012). And finally, (C) we illustrate how they are applicable to the design of new psychological treatments.

The picture we will outline sees humans as programmable biological machines whose psycho-social firmware is imprinted in attachment interactions, first and durably in the early years of life. Evolution “*designed*” this flexible mechanism to maximize fitness according to multiple selective pressures involved in the relationships with our primary caregivers. Attachment evolved as the motivational and data system responding to such pressures by allowing us to acquire corresponding implicit data – which we term attachment dimensions. Given the environmental cues conveyed by caregiving practices, their adaptive value is not limited to optimizing our primary relationships but extends to directing our reproductive strategies. Attachment dimensions address their adaptive goal by modulating a particular motivation. Somaticity modulates our tendency to comply with an external reference and affiliate with a group as an adaptive defense from the threat of contagious diseases. Nonetheless, if the context of our early imprinting does not match the later one, we can become excessively prone to compliance and more vulnerable to SA and EDs. The explicit consideration of the cognitive-evolutionary features of attachment dimensions entails relevant theoretical and methodological implications for evolutionary psychiatry and clinical psychology. The presented hypotheses bring them into focus with respect to somaticity, demonstrating how it complements social and neuropsychological views of specific disorders and can favor the design of new treatments.

Two essential remarks before going into the details. (R1) By suggesting that somaticity affects the vulnerability to SA and EDs (H2), we propose it to be one of the possible factors causing the onset and maintenance of these conditions, not the only causal factor. Consistently, we show how this *firmware-level* hypothesis complements the OT and ALT – more focused on the *software* (social context) and *hardware* (neural substrate) levels, respectively (*cf.* 5.2). (R2) Our ultimate objective is to integrate evidence from multiple sources – such as neuroscience, developmental, evolutionary, and clinical psychology – supporting H1-H2 to encourage their direct testing. We do not aim to prove them but only provide reasonable grounds for bringing them to the attention of future research.

## 2 Background

Our hypotheses on the evolutionary value (H1) and possible consequences (H2) of somatic caregiving programming rely on the following two premises P1 and P2.

### 2.1 Premise 1 (P1): the ontogenesis of self-consciousness

Self-awareness – i.e., our ability to be somehow and to some extent aware of ourselves – is a complex and layered ability that can be implicit or explicit (Rochat, 2015). What distinguishes the implicit-explicit forms of awareness is the instantiation of a self-representation in our working memory. Only when we become able to do that, we can be explicitly self-aware – namely, self-conscious (Carruthers, 2015). Ontogenetically, humans develop self-awareness in incremental stages from being purely implicit to being also explicit, showing the ability of self-consciousness at around 2 years and completing its maturation in the next 3 years (Rochat et al., 2012; Rochat, 2018) (Table 1). Each stage is marked by the emergence of new emotions, from more basic ones – such as happiness, sadness, anger, etc. – to more complex social emotions – such as shame, hubris, pride, and guilt (Ekman, 1992; Lewis, 2016).

**Table 1:** Developmental stages of self-representation.

	Developmental emergency	Self-awareness stage	Emotions experienced	Self-experience
1	0–7 months	**Implicit** (“I”)	happiness, sadness, anger, disgust, surprise	Sense of being differentiated from the rest
2	7–14 months	**“Post-implicit”**	fear	Sense of external co-experiencing
3	14–24 months	**“Pre-explicit”** (“Me”)	embarrassment, empathy, jealousy	Sense of internal co-experiencing
4	24–60 months	**Explicit (self-consciousness)**	shame, guilt, hubris, pride	Sense of social presence with standards and rules

We can identify 4 stages in the development of self-representation, from fully implicit to fully explicit self-awareness (i.e., self-consciousness).

Developmental research suggests that self-consciousness is linked to social consciousness and a strong tendency to social conformity (Rochat et al., 2012; Botto and Rochat, 2019) (*cf.* 4.2.1). The development of this ability at about 24 months of age is signaled by passing the mirror test (the child begins recognizing themselves in their reflected image) and by the emergent sensitivity to social standards and rules, showed by social emotions such as shame and guilt. The ontogenesis of self-consciousness corresponds to that of autobiographical memory, which concerns records of events experienced as personal (Howe, 2013).

Finally, our self-awareness is closely related to the representation of our body. In this regard, our brain processes spatial information using two different reference frames (Galati et al., 2010; Riva, 2012): (1) The **egocentric frame** corresponds to our experience in the first-person, where objects’ positions are referred to our own location. This representation is primarily underpinned by the ongoing construction of percepts from the current stimuli received by the body sensors (somatoperception). On the other hand, (2) the **allocentric frame** corresponds to the experience of an external viewer, an imaginary third-person who observes objects from a location in our external space. This representation mainly relies on our semantic knowledge built about the objects (somatorepresentation). In the case of one’s body, (1) the egocentric perspective is underpinned by one’s current, integrated perception of their bodily state, (2) the allocentric perspective is based on one’s knowledge of their body as a physical object. We develop the abilities to build ego- and allo-centric representations during the first years of life, following an ontogenetic path analogous to the one that takes us to experience self-consciousness and autobiographical memory, finally consisting of a six-representational ***Body Matrix***, whose highest level corresponds to the third-person conscious experience of one’s body related to social standards (Riva, 2017) (Table 2).

**Table 2:** Developmental stages of body representation.

	Developmental emergency	Body representation	Frame	Bodily experience
1	Birth	Body Schema	Egocentric	Sentience: Unconscious experience of one’s bodily sensations
2	0–6 months	Spatial Body	Egocentric	Location: Unconscious experience of one’s location
3	6–12 months	Active Body	Egocentric	Agency: Unconscious experience of one’s agency
4	18–36 months	Personal Body	Egocentric	Individuality: First-person conscious experience of one’s body
5	24–48 months	Objectified Body	Allocentric	Objectification: Third-person conscious experience of one’s body
6	48+ months	Body Image	Allocentric	Sociality: Third-person conscious experience of one’s body related to social standards

These six kinds of representation constitute the *Body Matrix*.

Therefore, our general “psychological” self-consciousness corresponds to our bodily self-consciousness and they are both built with reference to the social context.

### 2.2 Premise 2 (P2): developmental caregiving programming

Humans are an altricial species with a deeply-rooted social nature. Infants are evolutionarily prepared to attach – after a few months from birth – to specific *caregivers* who provide the physical and psychological context indispensable for survival (Bowlby, 1969/1982; Marvin et al., 2016). In the first years of life, these *attachment figures* allow children to develop and acquire fundamental psycho-social knowledge – attachment mental representations forming an Internal Working Model (IWM) (Sherman et al., 2015; Gagliardi, 2021). This information drives their social behavior, generating typical attachment patterns – also termed styles. Despite being more evident in childhood, we can be an “*attacher*” at any age, and attachment styles characterize us throughout our lifespan (Marvin et al., 2016; Mikulincer and Shaver, 2016). Moreover, the correspondence of parental styles to those of their children suggests the transmission of attachment characteristics from one generation to the next (Sette et al., 2015; Verhage et al., 2016) as confirmed by studies of adoptive families, which demonstrated the acquired nature of attachment (Groh et al., 2017; Raby and Dozier, 2019).

Attachment is underpinned by several dimensions (Fraley et al., 2015; Paetzold et al., 2015). The three basic ones – *disorganization*, *avoidance*, and *ambivalence* (here termed **α-dimensions**) – are acquired in infancy (0–2 years). Low levels of avoidance and ambivalence constitute, by definition, a secure attachment. Four additional dimensions – *depressivity*, *phobicity*, *somaticity*, and *obsessivity* (here termed **β-dimensions**) – were suggested to be acquired at preschool-age (2–6 years) (Guidano, 1987; Nardi and Bellantuono, 2008; Gagliardi, 2021). Therefore, attachment is induced by the caregiver’s operation and has a representational and dimensional nature. **Developmental caregiving programming** over multiple dimensions allows children to adapt to their caregivers. But the motivation to attach and the relevance of the acquired socio-psychological knowledge persist over the lifespan, suggesting attachment to be closely related to personality (Levy et al., 2015; Young et al., 2019). Consistently, the Attachment-Personality Theory (APT) (Gagliardi, 2021, 2022a) proposes the α- and β-dimensions to be a core part of our personality acquired through imprinting. This species-specific implicit (i.e., unconscious, non-verbal) data-acquisition process is characterized by (1) being preordained by evolution to occur first in early-life sensitive periods and (2) being particularly resistant to change later in life, when a change is possible. Imprinting corresponds to molding neural networks during early development to reliably serve their functions with constrained possibilities of later modification, if any (Knudsen, 2013; Di Giorgio et al., 2017). Given these characteristics, we adopt a basic *brain-computer analogy* and consider our attachment imprinting as ***firmware*** programming – producing durable but still changeable data – as opposed to the virtually unchangeable hardware (the neural substrate) and relatively easily modifiable software (explicitly acquirable knowledge).

The role of imprinting in attachment is supported by a substantial body of evidence from ethology (Salzen, 1967), psychology (Bowlby, 1969/1982; Troller-Renfree and Fox, 2017), and neurobiology (Roth et al., 2016; Perry et al., 2017; Sullivan and Opendak, 2020). Bird imprinting is rigid – i.e., normally non-reprogrammable – and limited to the caregiver’s identity. By contrast, human imprinting is much more flexible – offering the possibility of reprogramming after the sensitive period – and involves the acquisition of not only the caregiver’s identity but also additional evolutionarily-crucial information over several dimensions. Rats show a simpler version of this mechanism that was neurobiologically detailed (Roth et al., 2016; Sullivan and Opendak, 2020). In this species, the pup’s brain programming relies on olfaction and is sustained by maternal behaviors that activate a specialized attachment circuit, which includes the locus coeruleus, the olfactory bulb, and the anterior piriform cortex. It involves the release of norepinephrine, which enables imprinting when the level of corticosterone is low. This process occurs in a sensitive period of about 10 days after birth. However, a few days later, the mother can act as a *social buffer*, significantly reducing her pups’ stress response and reenabling imprinting (Landers and Sullivan, 2012; Perry and Sullivan, 2014). This programming is not limited to the acquisition of the caregiver’s identity. Pups use their mothers’ quality of care to orient their current and future lives, demonstrating rats evolved a primitive three-dimensional attachment similar to the human α-dimensions.

In our species, an early sensitive period for attachment was identified in infancy, between about months 2 and 24 (Troller-Renfree and Fox, 2017; Gee and Cohodes, 2021). However, after the first two years of life, parental care is still indispensable for the child’s survival and psychological development, and evidence suggests that our early sensitive periods extend to the preschool years and possibly until prepuberty (Perry et al., 2017; Luby et al., 2020). In particular, studies on human neurodevelopment found that different caregiving-related experiences at different timeframes correlate with the development of different brain areas (Rao et al., 2010; Luby et al., 2016; Teicher et al., 2018), pointing to the **preschool-age** as a **sensitive period** with respect to adverse childhood experiences and maternal support. Consistently with this evidence, the β-dimensions were suggested to be first imprinted in the preschool years, each in relation to a specific caregiving task – onto- and phylo-genetically – particularly relevant at this age (Gagliardi, 2021, 2022a). Among them, *somaticity* concerns the reflection of the child’s internal states – such as sensations, emotions, and representations – for self-regulation: a specific achievement of this age (Kochanska and Aksan, 2006; Holodynski, 2009). Through such a reflection, the caregiver supports the child’s ability to explicitly and autonomously identify and define their states, enabling self-regulation – i.e., the child’s ability to deal with social standards and expectations by regulating behavior and emotions appropriately (Boldt et al., 2020; Cassibba and Coppola, 2022). When the caregiver tends to define/impose rather than reflect the child’s states, the child remains uncertain about themselves and acquires the implicit information of not being able to self-define. They tend not to rely on their *somatoperception* and become over-dependent on their *somatorepresentation* and external references for regulation, primarily the caregiver (Guidano, 1987; Monteleone et al., 2020; Gagliardi, 2021).

In conclusion, during the first years of life, the child’s brain expects to be programmed by the caregiver, with domain-specific sensitive periods in infancy and preschool-age. Somaticity corresponds to one of these domains.

## 3 Hypotheses

Premises P1 and P2 lead us to hypothesize a causal relationship between early caregiving, the acquisition of durable (i.e., change-resistant) information, and the predisposition to develop certain mental conditions, in particular, with respect to somaticity.

**Figure 1:**
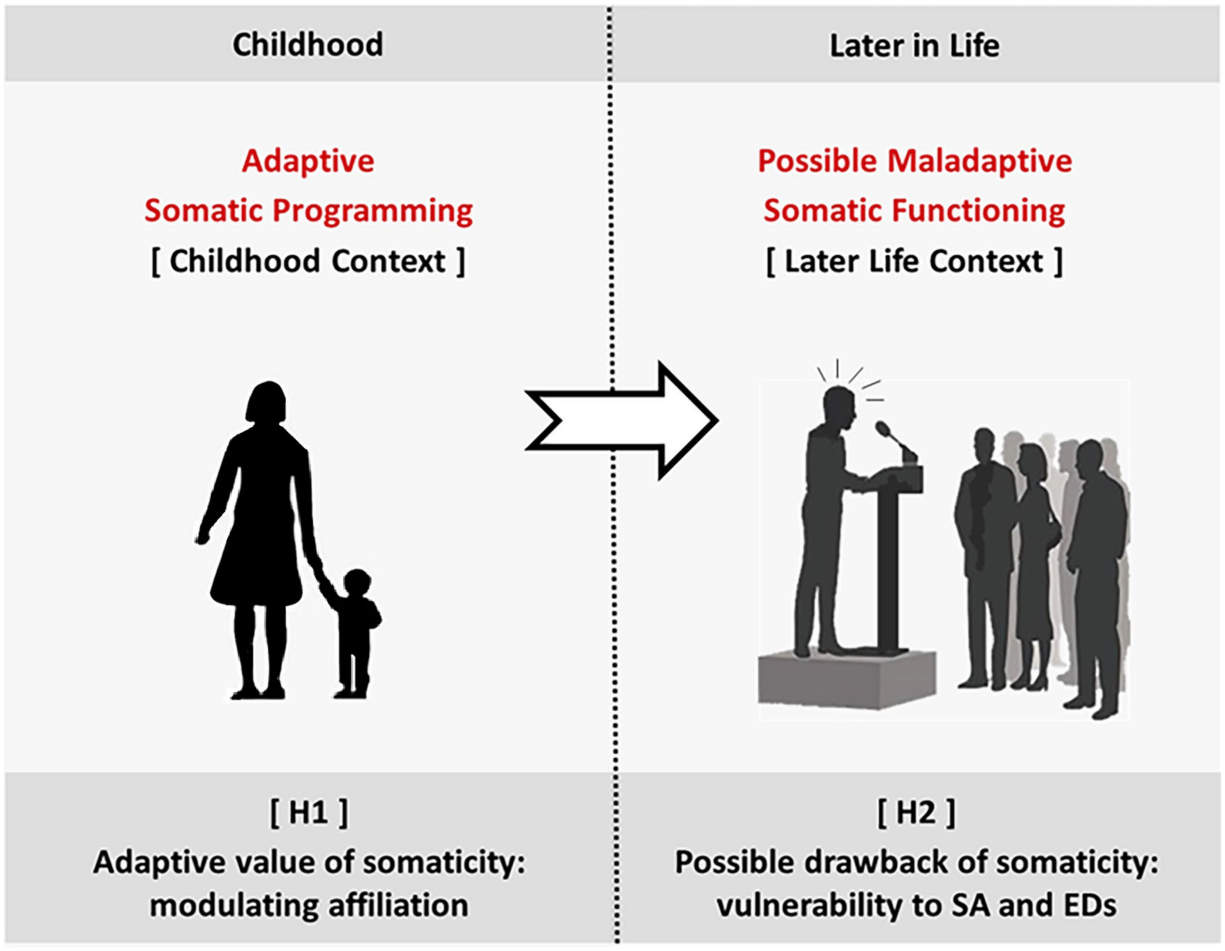
Our hypotheses. H1 [Adaptive value of somaticity]: During childhood, caregiving induces the child’s adaptive programming over several attachment dimensions. According to H1, somaticity provides optimal modulation of the tendency to comply and affiliate. H2 [Possible drawback of somaticity]: Later in life, if the context, for some reason, does not match the one for which somaticity was programmed, maladaptive somatic functioning may arise. H2 suggests vulnerability to SA and EDs.

Following P1, by the end of the second year, the child becomes self-conscious – i.e., able to have an explicit self-representation. This ability includes the possibility of consciously representing their own body and themselves in the social context – with the acquisition of social emotions, such as shame and guilt. In other words, at the beginning of the third year, the child can explicitly see themselves as a player in a social arena characterized by standards and rules. Following P2, since the child depends on the attachment figure(s) for survival, the preschool-age social context primarily consists of the caregiver(s), whose operation provides indispensable programming for the child’s developing brain. In the attachment exchanges, different caregiving features affect different aspects of the child’s development. In these years, one critical task of the caregiver is to support the child’s skills to integrate themselves into an expanding social network, where other peers and adults come into play. In this regard, the child acquires *somaticity*, the attachment dimension carrying implicit information on their ability to self-define and -regulate. Complying with social standards and expectations is indispensable to be accepted as part of a group (i.e., to affiliate). But at the same time, recognizing one’s internal states is essential to regulate the balance between one’s needs and the group affiliation requirements. On these grounds, we hypothesize that (Figure 1):

*H1*: Adaptive value of somaticity: Modulating affiliation.

The preschool-age acquisition of somaticity informs the child about how important it is to comply with external social references (primarily the caregiver). In other words, it sets the adaptive value of compliance in the current (socio-physical) environment. The higher this value, the higher the push to affiliate (with others in general).

*H2*: Possible drawback of somaticity: Vulnerability to social anxiety and eating disorders.

The acquired level of somaticity – i.e., the implicit information about the adaptative value of compliance – affects the vulnerability to Social Anxiety (SA) and Eating Disorders (EDs) later in life (given the close relationship of these conditions with our bodily and social experience).

## 4 Evaluation of the hypotheses

Our premises point out that human self-awareness is rooted in bodily experience (P1) and these two are closely related to attachment – in particular, to somaticity (P2). The more the caregiver does not confirm the child’s states, the more the child becomes somatic – i.e., uncertain of themselves and prone to conform to external references (Guidano, 1987; Monteleone et al., 2020; Gagliardi, 2021). Building on these considerations, we propose the two hypotheses H1-H2, which we evaluate here in turn. For the first, we will primarily rely on evidence-based evolutionary arguments. For the second, we will consider the psychological literature investigating the relationship between SA and EDs and (1) interoception – the component of somatoperception concerning inside inputs – and (2) parenting (the foundational elements of somaticity). But before doing that, we need to clarify the evolutionary-motivational properties of the attachment dimensions.

### 4.1 Attachment dimensions evolutionary-motivational properties

Early attachment theory established an evolutionary scientific framework focused on the survival role of attaching at the beginning of life (Bowlby, 1969/1982). On the other hand, the more recent life-history approach has stressed its value for reproduction in adulthood (Hertler et al., 2018; Szepsenwol and Simpson, 2021). Moreover, since attachment is about acquiring adaptation-essential data conveyed by multiple caregiving features, it is also a cognitive phenomenon. Within this **cognitive-evolutionary framework**, we discuss the adaptive value of the early acquisition of somaticity.

As discussed above (*cf.* 2.2), besides the caregiver’s identity, the infant-attacher gets first imprinted the information related to the three basic α-dimensions – disorganization, avoidance, and ambivalence. The acquired data concerns the caregiver’s frightfulness (disorganization), sensitivity (avoidance), and responsiveness (ambivalence). In other words, the infant stores implicit (non-verbal) information on how much the caregiver is trustworthy (meaning non-dangerous), prone to provide emotional care, and physically available when needed, respectively (Fraley and Spieker, 2003; Gagliardi, 2021). The brain operates as a control system driven by these dimensional representations (Petters, 2019; Gagliardi, 2022b), correcting action to reduce the discrepancy between what is currently experienced and the expectations provided by them. In other words, the dimensions are used as predictive models to optimize the interactions with the caregiver. This way, they play an essential survival role.

Additionally, life history theory stresses that every individual is structured to instantiate a reproductive strategy through their existence (Del Giudice et al., 2015). Consistently – given its relevance throughout life and, in particular, in romantic relationships (Fraley and Shaver, 2000; Mikulincer and Shaver, 2016) – attachment must play a role in such a strategy. In other words, attachment experiences must contribute to human **inclusive fitness** – i.e., gene preservation – by promoting future reproduction. Indeed, in the *Environment of Evolutionary Adaptedness* (EEA) (Bowlby, 1969/1982) – i.e., the context of human evolution (in terms of selective pressures) – caregiving practices generally corresponded to key environmental characteristics and, therefore, the acquired attachment dimensions were also crucial information on the quality of the (socio-physical) environment, which the child stored and later used for their reproductive strategy (Chisholm, 1996; Chester et al., 2012; Simpson and Belsky, 2016). The child’s adaptation to the caregiving features is also an adaptation to their environment. These features work as cues taken by the attachment dimensions to optimize mating strategies depending on the resources available. In fact, the limitation of resources imposes tradeoffs to reproduce successfully (i.e., maximize inclusive fitness), especially between mating and parenting (Beall and Schaller, 2019). This latter compromise leads to two possible mating strategies (Del Giudice et al., 2015; Simpson and Belsky, 2016): (1) **Slow strategy**. When the environment is generous with resources, one expects a longer life. Consequently, they tend to invest more in parenting and less in mating: delayed/less frequent reproduction, longer partnerships, fewer children but better prepared for the future (higher quality of offspring). (2) **Fast strategy**. When the environment is meager with resources, one expects a shorter life. Consequently, they tend to invest more in mating and less in parenting: earlier/more frequent reproduction, shorter partnerships, more children but less prepared for the future (lower quality of offspring).

#### 4.1.1 Avoidance and ambivalence

The scarcity of resources can correspond to two primary environmental characteristics (Ellis et al., 2009): (1) Harshness: measured in terms of *morbidity-mortality* – signaling how risky the environment is. (2) Unpredictability: measured in terms of *harshness* var*iation in space–time* – signaling how uncertain the environment is. These characteristics will lead the caregiver to adopt a fast strategy but show different caregiving features, inducing corresponding different attachment dimensions (Chisholm, 1996; Szepsenwol and Simpson, 2019): (1) In a harsh environment, the caregiver will focus on survival and appear to their children as ***unwilling to invest*** in them (i.e., unloving), thereby eliciting ***avoidance***. (2) In an unpredictable environment, the caregiver will be inconsistent (because often committed to survival priorities) and frequently appear to their children as ***unable to invest*** in them (i.e., unreliable), thereby eliciting ***ambivalence***. Therefore, the perceived unwillingness and inability to invest will induce (high levels of) avoidance and ambivalence, respectively (Chisholm, 1996; Chester et al., 2012; Simpson and Belsky, 2016). But in both cases, the significant difficulties faced by the child during development will make them try to grow as rapidly as they can and select a fast reproduction strategy to maximize inclusive fitness. On the other hand, when the environment is generous, the caregiver will generally be loving and reliable. As a result, caregiving will not induce (high levels of) avoidance and ambivalence, and children will adopt a slow strategy. In general, *“attachment phenomena and processes in childhood are systematically linked to the enactment of different reproductive strategies in adulthood”* (Simpson and Belsky, 2016, p.92). From a cognitive goal-belief perspective, this link corresponds to a tight connection between our intrinsic motivational systems (goals) (Liotti et al., 2017; Schaller et al., 2017; Brasini et al., 2020) and attachment dimensions (beliefs). Indeed, the specific adaptive advantage given by each dimension can be understood by considering its impact on a particular motivational system. From the above discussion, the case of avoidance and ambivalence appears clear: (1) An avoidant child experiences an unloving caregiver and deactivates the attachment motivational system – since no emotional care is expected (but *unwillingness to invest*) (Mikulincer and Shaver, 2016; McWilliams and Coveney, 2020). (2) An ambivalent child experiences an unreliable caregiver and hyperactivates the attachment motivational system – in an attempt to become the caregiver’s priority (given their current *inability to invest*) (Mikulincer and Shaver, 2016; McWilliams and Coveney, 2020).

#### 4.1.2 Disorganization

Compared to avoidance and ambivalence, ***disorganization*** is a more delicate case. Its evolutionary origin can be reasonably related to the high risk of parental abandonment, maltreatment, and infanticide in the EEA, which would occur for multiple reasons (Hrdy, 2009; Hrdy and Sieff, 2014; Reynolds et al., 2020): “*During our evolutionary history, when resources were scarce, when mothers had insufficient social support, or when children were spaced too closely together, if women were to have any surviving descendents they needed to favor some children over others. During particularly difficult times, that meant nurturing some children while abandoning others to die. Most at risk of abandonment were infants*” (Hrdy and Sieff, 2014, p.182). An infant would be neglected or maltreated for being perceived as having low reproductive value – i.e., low chances to survive and reproduce. The corresponding information stored by the survived disorganized child would be that of a ***dangerous*** caregiver, which would favor survival through the activation of the defense motivational system – eliciting the fight-flight-freeze-faint responses (Liotti et al., 2017; Steele, 2021).

The above evolutionary analysis of the three (basic) α-dimensions allows us to integrate the ontogenetic perspective with the phylogenetic one. In all cases, attachment provides a fundamental **adaptive advantage** by allowing for the early acquisition of implicit information that affects motivational activation (and overall dynamics), targeting a specific system: (1) Avoidance: attachment deactivation. (2) Ambivalence: attachment hyper-activation. (3) Disorganization: defense activation. The possibility of acquiring different dimensional levels according to the current context – as evident from the different degrees of possible avoidance, ambivalence, and disorganization (Fraley and Spieker, 2003; Hesse, 2008) – allows for modulating the pressure exerted by the acquired information and its motivational consequences. For example, a moderately risky/unpredictable environment will correspond to a moderate level of internalized avoidance/ambivalence and moderate pressure toward attachment de/hyper-activation.

**Figure 2:**
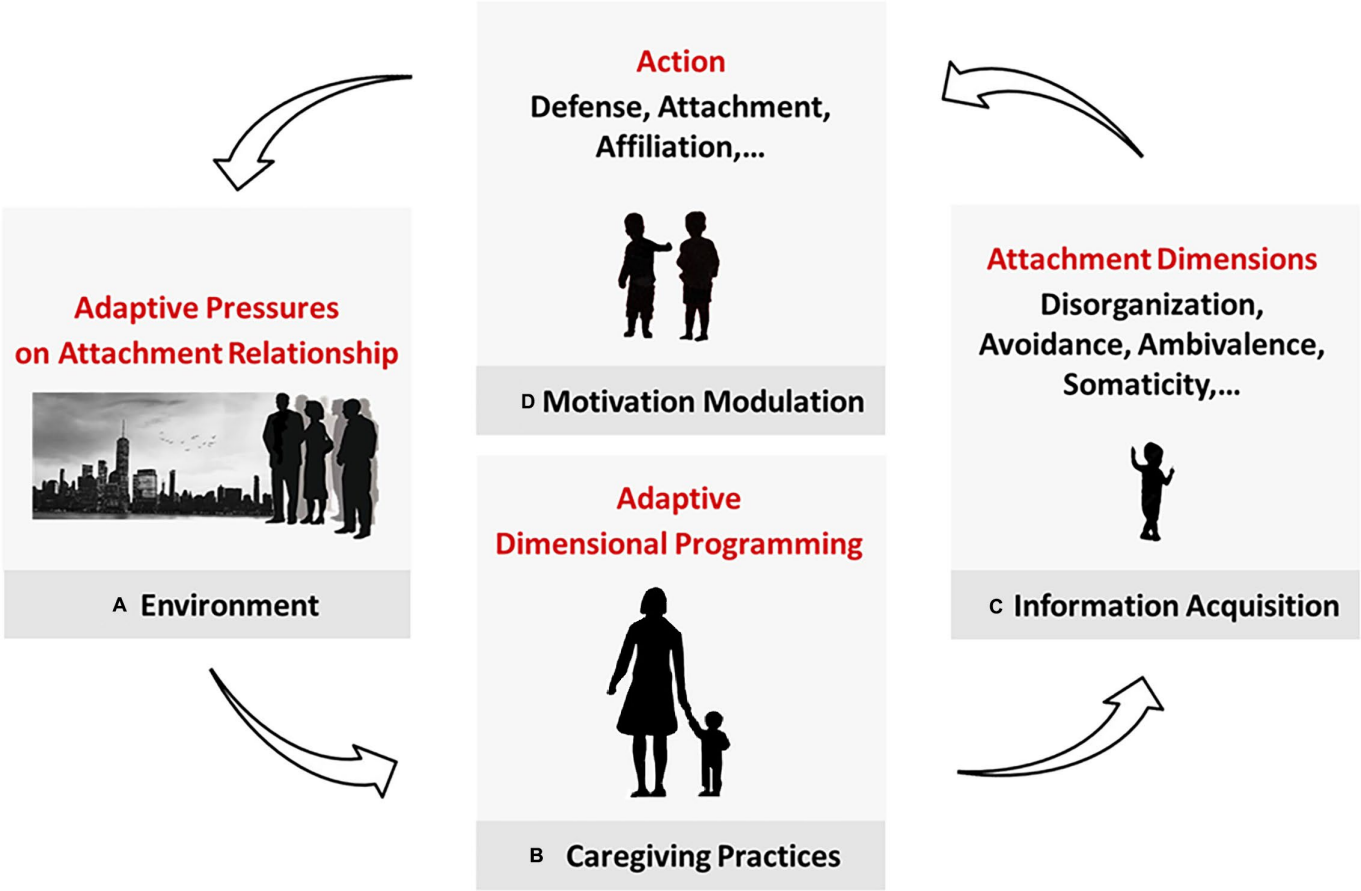
Acquisition and purpose of the attachment dimensions. **(A)** The environment poses adaptive pressures and affects **(B)** caregiving practices, which program the child’s attachment-related adaptation. **(C)** In attachment interactions, the caregiver conveys environmental cues that are translated into attachment data and **(D)** used to modulate motivation. Avoidance and ambivalence control (in different ways) the motivation to attach. Disorganization affects the defense system. We propose here that somaticity concerns our tendency to affiliate. (In future works, we will suggest phobicity, depressivity, and obsessivity modulate self-care, ranking, and caregiving, respectively). This mechanism allows the child to adapt to their caregiver first and their more general environment progressively in life.

The same rationale should apply to the β-dimensions, considering that each dimension must have provided a distinct advantage to be selected. The attachment system is an evolutionarily preordained device that is programmed according to the contingent requirements to flexibly solve crucial adaptive problems. It does so by **acquiring information** and using it **to modulate motivation** in specific ways (Figure 2). We now consider the case of somaticity.

We evaluate H1-H2 in the following two sections (*cf.* 4.2, 4.3) and discuss our results in the subsequent one (*cf.* 5).

### 4.2 Evaluation of H1. Adaptive value of somaticity: modulating affiliation

The outlined evolutionary-motivational framework indicates that each attachment dimension should allow for modulating the tendency to activate a specific motivation, thereby providing a crucial adaptive advantage. Therefore, the evaluation of H1 needs to consider (A) the motivation related to somaticity and (B) the advantage given by modulating the propensity to activate it.

**Somaticity** has been defined as the attachment dimension linked to the caregiving task of supporting the child’s ability to define their own internal states. The more the caregiver does not confirm the child’s states, the more the child will seek an external definition (Guidano, 1987; Monteleone et al., 2020; Gagliardi, 2021). Accordingly, H1 suggests that somaticity provides a fundamental **adaptive advantage** by allowing for the early acquisition of implicit information about the necessity to comply with external social references (primarily the caregiver), which implies **affiliation**. The caregiver programs the child with implicit knowledge – i.e., durable, low-level data – about the value of compliance to social standards and expectations by encoding it in the level of the child’s uncertainty. In a given context, this flexible mechanism should allow the individual to find the *most effective balance between threat protection and openness to opportunity* (Lewis et al., 2017). When complying is more relevant for survival, one should feel more compelled to affiliate. But they should feel freer to express themselves and pursue other motivations in a less critical context. The threat involved needs to be specified. Accordingly, to evaluate this hypothesis, we consider next:

1. If affiliation is actually an intrinsic motivation co-evolved with attachment (*cf.* 4.2.1).
2. And in this case, the threat-opportunity balance related to affiliation (*cf.* 4.2.2).

#### 4.2.1 The intrinsic motivation to affiliate

Concerning the motivation to affiliate, it is worth noting that our species evolved most of its psychological characteristics during the Pleistocene, while living as nomad hunter-gatherers in small bands of 30–50 members (Starratt and Shackelford, 2010; Zlatev, 2014). The primary evolutionary period, critical for social evolution, is believed to cover between *ca.* 1.8 Mya and 10 Kya from *Homo Ergaster* to *Erectus* to *Heidelbergensis* to *Sapiens*, before the transition to settled life based on plant/livestock farming – around 10 thousand years ago indeed. At the onset of the process, cooperative breeding – also known as alloparenting – was selected as the most adaptive rearing practice (Hrdy, 2009; Zlatev, 2014). Mothers increased the chances of their children’s survival by relying on the additional caregiving provided by other band members. Strong multidisciplinary arguments suggest that this adaptive solution propelled cognitive, emotional, and motivational enhancements that led first to the evolution of intersubjectivity and then the co-evolution of social norms and language (Hrdy, 2009; Tomasello et al., 2012; Zlatev, 2014). These arguments point to a Multi-Level Selection process where affiliation to a group has allowed the specific higher-level human features to evolve, transcending the initial purely individualistic purposes. In our species, belonging to a group originated and co-evolved with attachment and is rooted in sharing mental states. *The tendency to affiliate has been evolutionarily hard-wired as an intrinsic motivation*, implying numerous ways of favoring one’s group over others (Liotti et al., 2017; Schaller et al., 2017). This view is strikingly confirmed by the minimal group phenomenon, widely studied in social psychology, where individuals tend to affiliate with a group by relying on randomly-assigned common irrelevant characteristics – such as an arbitrarily given group letter or a t-shirt of the same color (Tajfel and Turner, 2004; Dunham, 2018). Belonging to a minimal group is enough to favor members of one’s group (the ingroup) over another (the outgroup). And this phenomenon starts to be observed in childhood with precursors even in infancy.

Attachment and sociality are the phylogenetic roots of the most uniquely human characteristics, such as intersubjectivity and culture. Consistently, evolution hard-wired our brains with the intrinsic motivation to affiliate with a group.

#### 4.2.2 The threat-opportunity balance related to affiliation

The **Parasite-Stress Theory** (PST) (Thornhill and Fincher, 2014b, 2015) suggests how somaticity can have evolutionarily affected a balance between threat and opportunity. The PST refers to pathogenic agents that cause infectious diseases as “*parasites*’ and focuses on non-zoonotic illnesses, i.e., those a human can receive from another human (directly or through a vector) – as opposed to zoonotic illnesses, i.e., those a human can receive from a non-human animal (Thornhill et al., 2010; Fincher and Thornhill, 2012; Thornhill and Fincher, 2014a). The theory proposes that avoiding such diseases has been a primary drive in the evolution of uniquely-human social and psychological characteristics. In fact, our species evolved a specific **Behavioral Immune System** (BIS) (Schaller, 2006; Thornhill and Fincher, 2014b) – consisting of behavioral as well as emotional and cognitive adaptations – to deal with pathogenic threats. In this regard, the most relevant factor was the *spatial–temporal distribution of pathogens*, which brought about two kinds of evolutionary solutions. (1) First, a group living long enough in the same infectious context would adapt to its pathogens. As a result, their BIS provides tendencies to manage evolutionarily-known pathogenic threats, such as those related to individual hygiene. (2) Second, for a group, interactions with groups from other regions would imply the transmission of diseases against which the group evolved no defenses. As a result, their BIS includes psychological mechanisms devoted to distinguishing and protecting the ingroup against outgroups to limit parasitic threats. Overall, parasitic pressures made us evolve mechanisms toward “***assortative sociality***,” whose components are “*(1) limited dispersal for reproduction from the natal locale* [i.e., philopatry]*, (2) in-group favoritism* [i.e., ethnocentrism]*, and (3) out-group dislike and avoidance* [i.e., xenophobia]” (Fincher and Thornhill, 2012, p.261). In other words, humans have built-in tendencies toward ingroup cohesion and outgroup wariness.

Here, we report some of the numerous studies supporting the PST and involving variables particularly relevant to our hypotheses. Consistently with the theory, the analysis of geo-historical data found that the macro-social polarities **collectivism–individualism** and **conservativism-liberalism** are strongly correlated to the rate of infectious diseases (Fincher and Thornhill, 2012; Thornhill and Fincher, 2014a; Bennett and Nikolaev, 2020). More collectivist and conservative (/individualistic and liberal) societies are tightly linked to a higher (/lower) presence of infectious diseases. Hence, higher (/lower) infection rates correlate with adopting more collectivistic and conservative (/individualistic and liberal) value systems. Coherently, members of groups more exposed to pathogenic threats manifest higher **conformity** to social norms (Murray and Schaller, 2012, 2016). Such compliance tends to reduce the risk of infection – for example, through established practices regarding hygiene and nutrition. The correlation between perceived vulnerability to disease and compliance attitude was also found when manipulating infection risk experimentally: The higher the vulnerability/risk, the higher the compliance (Murray and Schaller, 2012; Wu and Chang, 2012). In this regard, a particular sign of conformity is provided by the extent to which group members adhere to religious precepts. As expected, such adherence was stricter when the pathogenic threat was higher (Fincher and Thornhill, 2012; Thornhill and Fincher, 2014c). Consistently, innovation – defined abstractly as the development and support for new ideas and artifacts – was shown to be correlated with low infection rates (Murray, 2014; Bennett and Nikolaev, 2020). Regions historically higher in pathogen threat were those less innovative today, according to multiple measures. In accordance with the PST, this relationship was mediated by individualistic values, supporting the idea that innovation is driven by liberalism and nonconformity. Finally, the analysis of data from numerous contemporary-world countries also allowed linking parasitic stress to **family ties** (Fincher and Thornhill, 2012; Thornhill and Fincher, 2014a). Again, predictably, stronger familial cohesion corresponded to higher infection rates. Overall, the investigation of these variables confirms the hypothesis on the role played by infectious diseases in the evolution of human socio-psychology. Avoiding socially transmittable illnesses pressed our ancestors toward assortative sociality – with its pro-ingroup/anti-outgroup tendencies and practical consequences.

It is noteworthy that *conformity* and *family ties* are tightly related to the definition of *somaticity* and are also linked to SA and EDs – the mental conditions we suggest being possible clinical manifestations of somaticity. For example, socially anxious individuals were shown to have a stronger tendency to comply with the group (affiliation) compared to non-anxious controls (Feng et al., 2018). The same inclination was revealed in people suffering from eating disorders by their enhanced fear of being judged (Butler et al., 2023).

Having identified the principal selective pressure related to affiliation, we can discuss the suggested modulating role of somaticity. In this regard, we need to consider that (Thornhill and Fincher, 2014b):

1. In the EEA, infectious diseases were variable in space and time, with different timescales requiring different kinds of adaptations – from the genetic to the high-cognition level. On one extreme, stably inhabiting a given area – a malarial region, for example (Kwiatkowski, 2005) – would favor the selection of some genes working against the infections that were characteristic of that area (“*hardware*” solution) (Barreiro and Quintana-Murci, 2010). On the other extreme, frequently moving to a different location would rely on the high-level cognitive skills necessary to quickly find and adopt new and safer practices (“*software*” solution). Arguably, the most common scenario was one in between. Namely, our ancestors were most often exposed to conditions persisting for one generation to the next, which would be effectively faced by evolving an early-life implicit and durable learning mechanism (“***firmware***” solution).
2. For the ancestral ingroup, being open to an outgroup simultaneously implied possible costs – facing new pathogenic agents without built-in defenses – and benefits – accessing new resources, which could consist of ideas, tools, and mating partners, for example. Therefore, finding the *optimal compromise between possible costs and benefits* was evolutionarily essential.

Given these factors, we expect evolution to have provided our species with a mechanism allowing us to adapt to intergeneration-persistent pathogenic conditions and help regulate our openness to outgroups. Attachment – i.e., early-life programming from caregiving – appears to be the optimal solution. It ensures the implicit acquisition of reliable information (compatibly with cognitive development) while being flexible enough to adjust its parameters according to the contingent environmental conditions (*firmware* solution). And somaticity matches the specific affiliation problem posed by pathogens. More specifically, this mechanism would work as follows. The degree of environmental parasite stress affects caregiving practices. The higher the threat, the more the caregivers impose normative group behaviors on their children, thereby often overriding their immediate needs and disconfirming their internal states. This attitude is captured by somaticity, which informs the child about the relevance of complying with external references – first their caregivers and then larger parts of the ingroup. The acquisition of implicit, durable information matches the evolutionarily frequent occurrence of environments persisting from one generation to the next. Thus, parasite stress justifies somaticity and its adaptive function of modulating our tendency to affiliate on an intergenerational time scale.

### 4.3 Evaluation of H2. Possible drawback of somaticity: Vulnerability To social anxiety and eating disorders

Our above discussion supports the adaptive function of attachment and the role played by its somatic dimension in the modulation of affiliation. Somaticity corresponds to the implicit information we acquire about the need for compliance with external references. We bodily encode this knowledge in the degree of uncertainty about our internal states (Guidano, 1987; Monteleone et al., 2020; Gagliardi, 2021), which are the object of our interoception – i.e., the neural processing of our internal (bodily) signals, consisting of sensing, integrating, and interpreting them. Therefore, in the following two sections, we examine the relationship between interoception and parenting style (the foundational elements of somaticity) and SA (*cf.* 4.3.1) and EDs (*cf.* 4.3.2).

Before proceeding, we need to note that the operation of interoception can be assessed on multiple levels, and we will focus on the aspects most relevant to SA and EDs – accuracy and awareness – that can be defined as follows (Garfinkel et al., 2015). **Interoceptive Accuracy** (IAc) is one’s ability to detect and report their internal state in terms of a reference parameter. The most common method to measure IAc is the Heartbeat Counting Task, consisting in counting the number of one’s heartbeats in a given time. On the other hand, **Interoceptive Awareness** (IAw) is one’s metacognitive ability concerning their interoception – in particular, the interpretation of one’s bodily signals. IAw is most often measured through self-reports.

#### 4.3.1 Interoception, parenting style, and vulnerability to social anxiety

Social Anxiety Disorder (SAD) concerns situations in which the individual feels they will be exposed to others’ scrutiny (APA, 2013). According to the well-established Clark-Wells cognitive model of SAD (originally termed Social Phobia), in these situations, the socially anxious tends to focus on themselves, have negative expectations, and experience anxiety (Leigh and Clark, 2018). Consistently, the self-image one thinks to give – with its psychological and physical aspects – is the condition’s major component. What specific element becomes relevant for the individual depends on their past experiences and the context where the action unfolds. One might focus on how they look confident while talking, another on whether their appearance meets the social standards, for example. We will now demonstrate that, in all cases, interoception is crucial. Then, we will consider the parenting style most closely related to the development of SA.

##### 4.3.1.1 Interoception and SA

Psychological investigation shows that socially anxious individuals are affected by anomalous interoceptive functioning. Two studies where children (9–13 years old) were exposed to adults’ judgment when completing a task found those high in SA had deficient interoceptive awareness (IAw) with subjective evaluation not corresponding to objective perceptive abilities, but unaltered interoceptive accuracy (IAc) (Schmitz et al., 2012; Asbrand et al., 2020). As expected, these children were characterized by excessive attention to bodily signals. In similar conditions, when socially anxious adults were told they would perform a task and be evaluated, this social stimulus had a higher activation effect on them than on the less anxious individuals (Stevens et al., 2011). In this case, the anxious ones also showed increased IAc and rated their anxiety higher. In accordance with these results, adults high in SA who were given random false feedback of accelerated heartrate while performing a task were found to have enhanced neural processing of cardiac activity related to such feedback (Judah et al., 2018). This finding confirms the bodily hyper-focus of socially anxious individuals and is consistent with their increased IAc in social situations. However, when a task involving negative social feedback (faces expressing anger or fear) was performed during an fMRI scan, subjects with SAD showed lower IAc and anomalous cardio-regulatory functioning (Gaebler et al., 2013) – possibly due to excessive activation in this particular experimental condition. In accordance with their physiological hyper-activation in social situations, these participants reported an increased tendency to self-focus and suppress emotions, consistent with deficient IAw. These studies demonstrate that – while IAc may be affected by the activation level – socially anxious individuals excessively react to social stimuli and have impaired IAw.

An additional point of interest concerns the role of emotions. Given their physiological/interoceptive component, we expect difficulties in one’s emotional identification and processing to be related to SA. This hypothesis is confirmed by the correlation found between alexithymia^1^ and SA (Lyvers et al., 2019; Panayiotou et al., 2020).

In accordance with the Clark-Wells cognitive model of SAD (Leigh and Clark, 2018), overall, these studies point to a **lack of interoceptive awareness**. In social situations, SA individuals excessively focus on how others see them and are hyper-sensitive to signs of rejection. Their bodily signals become part of their image and are interpreted negatively. Interoception is subjected to a cognitive bias related to social stimuli processing (excessive focus, hyper-sensitivity) – affecting, in particular, interoceptive awareness (impaired interpretation of internal signals). This cognition-driven alteration of interoceptive functioning is consistent with the proposed somatic knowledge acquisition and consequent hyper-tendency to comply and affiliate. The socially anxious is worried about not meeting the requirements for group acceptance.

##### 4.3.1.2 Parenting style and SA

Finally, to evaluate the involvement of somaticity in the vulnerability to SA, we examine the parental practices linked to this condition. The relevance of caregiving to SA is indirectly suggested by its modest heritability and early onset. A recent genetic study on a large sample estimated the heritability of SA to be around 15% (Stein et al., 2017), although previous research on SAD has been significantly inconsistent (Stein and Gelernter, 2014; Moreno et al., 2016). Concerning its onset, SAD typically arises in adolescence but often in childhood, with cases reported since the early school years (Chavira and Stein, 2005; Lijster et al., 2017). Literature investigating the relationship between caregiving and the development of SA points to the role of an intrusive parental attitude – often referred to as “***psychological control***”, “*overcontrol*”, or “*overprotection*”. This kind of control is conceptualized as a psychologically manipulative practice that undermines the child’s autonomy (Gulley et al., 2014; Hastings et al., 2019). Gómez-Ortiz et al. (2019) measured six dimensions of parental practices and children’s SA on a sample of 2,060 adolescents aged 12–19, finding that psychological control – instantiated by “*manipulative and intrusive strategies such as inducing a feeling of guilt or withdrawing their affection*” (Gómez-Ortiz et al., 2019, p.125) – was the parental practice most highly correlated to SA. We report here some other representative studies that supported this significant relationship by using different methods and tools. In their longitudinal study from infancy to adolescence, Lewis-Morrarty et al. (2012) defined overcontrol as excessive control of the child’s natural behavioral or mental propensions. They operationalized this concept to apply it to their observations of mother–child interactions, identifying two degrees of overcontrol: (1) “*moderate*” – when the parent “*verbally dominates the conversation or the play activity, directing the child’s attention away from his/her interests and/or excluding the child from participation*,” and (2) “*outright*” – when the parent gives “*frequent unnecessary and restrictive instructions*” and/or performs “*physically controlling behaviors that change or stop the child’s play*” (Lewis-Morrarty et al., 2012, p.5). The authors found that the level of maternal overcontrol observed in childhood (age 7) was related to the severity of SA symptoms reported in adolescence (age 14–17). Similarly, Norton et al. (2022) – by relying on participants’ self-reports – found that overcontrol was the parental style correlating the most to adult SAD emotional states. Evident behavioral control – observable, for example, in parent–child play interactions – is underpinned by a more subtle psychological control, consisting in manipulating and inhibiting thoughts, emotions, and activities and resulting in impairing the child’s autonomy. In general, the literature identifies a parenting style characterized by critical rejection, overinvolvement/intrusiveness, and overprotection as related to the development of SA (Gulley et al., 2014; Asbrand et al., 2017). Among these features, critical rejection – instantiated by behaviors expressing disapproval or dismissal of the child’s own expressions – is especially relevant to the development of SA (Mills and Rubin, 1998; Gulley et al., 2014). Receiving such a parental response is related to feeling ashamed, an emotion central to SAD (Hedman et al., 2013; Norton and Abbott, 2017).

Hence, **parental psychological control** was found to be linked to the development of SA in children. All the reported controlling practices imply the caregiver’s disconfirmation/definition of the child’s internal states suggested to induce somaticity. Moreover, the shame of the socially anxious is a sign of failure at attempting to be accepted/compliant, which also characterizes the somatic. In conclusion, evidence suggests SA to be linked to impaired IAw and parental psychological control, thereby supporting H2.

#### 4.3.2 Interoception, parenting style, and vulnerability to eating disorders

EDs include the mental conditions directly related to food practices. Here, we will consider the most relevant: **Anorexia Nervosa** (AN), **Bulimia Nervosa** (BN), and **Binge Eating Disorder** (BED). Their primary characteristics can be concisely recapitulated as: food intake restriction leading to significantly low weight (AN), recurrent episodes of binge eating with compensatory behaviors (BN), and recurrent episodes of binge eating without compensatory behaviors (BED), respectively (APA, 2013). Some forms of EDs do not fit any specific category and can be referred to as unspecified (UED). Interestingly, a disorder related to the excessive intake of food leading to significantly high weight is currently not present in the main psychiatric classifications. EDs are clinical conditions characterized by extreme disordered eating – a broader category of issues including unhealthy but still subclinical eating behaviors (e.g., dieting and overeating), often preceding an ED (Rohde et al., 2014; Stice et al., 2016). The Body Mass Index (BMI) is the standard parameter used to assess one’s nutritional condition in terms of weight (in kilograms) and height (in meters) – specifically, BMI = weight/height^2^ (the healthy range is 18.5 kg/m^2^ ≤ BMI < 25 kg/m^2^; a BMI ≥ 30 kg/m^2^ is considered obesity). EDs also have a close connection to body image, and the focus on appearance to others links them to SA (Levinson et al., 2018). Finally, it is worth noting that they are much more common among females than males (Galmiche et al., 2019). As with SA, we will first demonstrate the primary role of interoception in EDs and then consider the parenting style related to their development.

##### 4.3.2.1 Interoception and EDs

Psychological research suggests **deficient interoceptive awareness** (IAw) to be central in all EDs (Martin et al., 2019; Brown et al., 2020). A meta-analysis – comprising 41 studies with an overall sample of 4,308 ED and 3,459 healthy subjects – demonstrated that the individuals suffering from AN, BN, or BED and those who recovered from AN and BN had significantly impaired IAw compared to healthy controls (Jenkinson et al., 2018). A meta-analytic work on the relationship between interoception and BMI – considering 87 articles and 10,425 participants – found that higher overall interoceptive deficits (in particular, lower accuracy, IAc) were associated with higher BMI (Robinson et al., 2021a). Consistently, individuals affected by overweight or obesity showed poorer interoception compared to healthy controls. Next, we provide some more details on AN, BN, and obesity.

**[AN]** Despite some controversial results, data overall suggest that AN does not impact IAc significantly (Lutz et al., 2019; Teaford et al., 2021). On the other hand, studies consistently reported impaired IAw in anorexics, with increased distress and confusion in response to interoceptive signals (Fassino et al., 2004; Eshkevari et al., 2014; Lutz et al., 2019). Coherently, measures of neural activity in AN subjects showed hyper-elaboration of internal signals (Kerr et al., 2016; Lutz et al., 2019). Anorexics appear to have no impairment in detecting bodily signals but rather in giving them meaning with consequent distress and confusion. **[BN]** As with AN, studies on BN point to unaltered IAc (Eshkevari et al., 2014; Pollatos and Georgiou, 2016) and deficient IAw (Fassino et al., 2004; Eshkevari et al., 2014; Pollatos and Georgiou, 2016). And in line with difficulties in interpreting interoceptive signals, BN and BED subjects use binge eating as a dysfunctional strategy to regulate their emotions (Leehr et al., 2015; Meule et al., 2021). **[Obesity]** On the other hand, conversely to AN and BN, data indicate that overweight and obese individuals have a reduced IAc (Herbert and Pollatos, 2014). Coherently, multiple studies found a negative correlation between BMI and IAc (Herbert et al., 2013; Robinson et al., 2021b). However, similarly to AN and BN, obesity was shown to be linked to impaired IAw (Fassino et al., 2004; Willem et al., 2019). EDs and obesity share a **lack of interoceptive awareness**, which primarily consists in mistrusting one’s internal signals (Willem et al., 2019; Brown et al., 2020), hence, in their impaired interpretation. And these conditions were also linked to alexithymia (Fernandes et al., 2017; Westwood et al., 2017) and emotion regulation deficits (Fernandes et al., 2017; Prefit et al., 2019).

In conclusion, evidence suggests that cognitive biases – in particular, uncertainty over one’s sensations and emotions – compromise IAw, favoring the development of eating-related conditions and associated alexithymia and emotion regulation difficulties. Such uncertainty about one’s internal states – and consequent hyper-reliance on external references – are what attachment-acquired somatic knowledge is suggested to convey. The caregiving practices linked to this acquisition are the focus of the next section.

##### 4.3.2.2 Parenting style and EDs

To complete our evaluation of the involvement of somaticity in the vulnerability to EDs, we look now at the characteristics of parenting connected to these conditions.

Interestingly, epidemiological research usually indicates a moderate-to-strong genetic influence on EDs, primarily relying on twin studies – which provided heritability estimates in the ranges of 28–74% for AN, 54–83% for BN, and 39–57% for BED (Thornton et al., 2011; Bulik et al., 2016). Nonetheless, multiple reasons question the reliability of twin studies in general and on mental disorders in particular. In fact, they are based on several delicate assumptions and usually relatively small or non-disorder-specific datasets. Their most critical presupposition is the “*equal-environment assumption*”, according to which two identical (monozygotic) twins experience the same degree of environmental similarity – e.g., parental style and peer influence – as two non-identical (dizygotic) ones. Multiple studies demonstrated that this hypothesis – which results in estimating environmental impacts as genetic – is invalid since identical twins are significantly more likely to share the same environment and have the same experiences than non-identical ones (Joseph, 2013; Burt, 2015; Moore and Shenk, 2016). The proven connection between parental styles and EDs – examined below – is consistent with the need to downsize current heritability estimates.

Similarly to SA, EDs typically have onset in adolescence or early adulthood but can occur in childhood (Schaumberg et al., 2017; Favaro et al., 2019). Consistently, a large corpus of evidence suggests that parenting styles play a primary role in the vulnerability to EDs or the more general DE. In particular, ***parental psychological control*** (PPC) was identified as having a specific influence on children’s eating behaviors. Barber (1996) defined PPC as “*control attempts that intrude into the psychological and emotional development of the child”* (Barber, 1996, p. 3,296). The author indicated 6 behavioral categories instantiating PPC – (1) constraining verbal expressions, (2) invalidating feelings, (3) personal attack, (4) guilt induction, (5) love withdrawal, and (6) erratic emotional behavior – and developed a self-report to measure it – the Psychological Control Scale-Youth Self-Report (PCS-YSR) (Barber, 1996). The studies that used the PCS-YSR to explore PPC and DE consistently demonstrated the association between the two (Reilly et al., 2016; King et al., 2022), reporting the specific correlations between *invalidating feelings* and under-eating and between *personal attack* and over-eating (Romm et al., 2019; Romm and Alvis, 2022). The correlation between PPC and DE was confirmed by studies using other tools – such as the Parental Bonding Instrument (PBI) (Parker et al., 1979) and the Invalidating Childhood Environment Scale (ICES) (Mountford et al., 2007). The PBI-measured “*overprotection*” – “*defined by control, overprotection, intrusion, excessive contact, infantilization and prevention of independent behavior*” (Parker et al., 1979, p.8) – was linked to the development of EDs and obesity (Tetley et al., 2014; Amianto et al., 2021). Similarly, invalidating parenting measured by the ICES – whose key characteristic is “*the non-recognition of the actual state of the child*” (Mountford et al., 2007, p.49) – was associated with subclinical DE and EDs (Gonçalves et al., 2018; Sivanathan et al., 2019). Crucially to our evaluation, all these instruments identify parental behaviors aimed at obtaining the child’s compliance, instantiating the disconfirmation/definition characterizing somaticity.

The presented studies demonstrate the connection between PPC – a central feature of somaticity – and EDs. Therefore, we can conclude that – as with SA – evidence suggests EDs to be linked to impaired IAw and PPC, thereby supporting H2. It is also worth noting that SAD and EDs are highly comorbid (Kerr-Gaffney et al., 2018; Levinson et al., 2018) – SAD being more frequent than any other anxiety disorder in EDs (Swinbourne et al., 2012). This coexistence is consistent with the possible common somatic etiology proposed in this work.

## 5 Discussion

In this work, we put forward two hypotheses (H1-H2) on the developmental-evolutionary origin of our sociality and its possible psychopathological consequences. We then evaluated H1-H2, addressing the following three essential points.

*(1) Relevant attachment properties (cf. 4.1).* We started our evaluation by discussing the evolutionary properties of attachment, focusing on its adaptive function as both a motivational and knowledge system. When adopting an evolutionary perspective, attachment dimensions appear to work as an adaptive mechanism for **modulating intrinsic motivations** in favor of the optimal balance between threat and opportunity. The key elements of this mechanism are the following. (1) Caregiving consists of several evolutionarily fundamental tasks/features. (2) Each feature corresponds to an attachment dimension. (3) Each dimension allows us to collect specific implicit, durable information. (4) The collected data sets our sensitivity to a fundamental adaptive problem. (5) This sensitivity affects the way we use our motivational systems, i.e., our motivational dynamics. Therefore, attachment dimensions optimize the current relationship with the caregiver but – importantly – also the fitness to the future context through the environmental cues conveyed by the caregiver’s action. In other words, our caregiver programs (part of) our overall adaptation. Avoidance and ambivalence are influenced by the caregiver’s sensitivity and responsiveness and steer the motivation to attach toward deactivation and hyper-activation, respectively. On the other hand, disorganization is affected by the caregiver’s frightfulness and involves the defense system.

*(2) Adaptive value of somaticity [H1] (cf. 4.2).* As an attachment dimension, somaticity is expected to act as a motivational modulator. Consistently, our analysis first identified affiliation as an intrinsic motivation rooted in alloparenting and leading to intersubjectivity and other uniquely human characteristics (*cf.* 4.2.1). Then, we turned to the specific threat-opportunity balance problem related to our tendency to affiliate and illustrated the possible ancestral link between this proclivity and somaticity (*cf.* 4.2.2). In other words, we suggested the evolutionary reason why we acquire from caregiving a certain degree of interoceptive uncertainty, which affects our tendency to comply and affiliate. As the Parasitic Stress Theory suggests, the presence of pathogens had a crucial impact on our ancestors’ social life. More openness to outgroups could increase access to vital resources but, at the same time, exposure to lethal diseases. Accordingly, we proposed **pathogen threat** as the selective pressure determining the acquisition of a somatic attachment dimension. Since, ancestrally, the level of pathogenic risk frequently persisted from one generation to another, evolution was favored by passing implicit, reliable, and durable information on this critical environmental feature to the offspring. Somaticity is the ***firmware* solution** that evolution selected for the parasitic-social problem.

*(3) Possible drawback of somaticity [H2] (cf. 4.3).* To examine the involvement of somaticity in the vulnerability to Social Anxiety (SA) and Eating Disorders (EDs), we looked at the relationship between interoception and parenting style – foundational aspects of somaticity – and SA (*cf.* 4.3.1) and EDs (*cf.* 4.3.2): **[1]** Interoception is an essential component of these conditions. In both cases, the psychological literature points to **cognitive biases affecting interoceptive awareness** (IAw), with an impaired interpretation of internal signals. **[2]** Parental practices are also tightly related to SA and EDs. While heritability influences these disorders to an extent that still needs to be precisely estimated, parenting styles play an important role in their etiology. In particular, **parental psychological control** (PPC) emerged as the caregiving feature regularly associated with the subsequent development of both SA and EDs. This parental manipulative and intrusive attitude – resulting in inhibiting or overriding the child’s natural emotional and cognitive expressions – fully matches the defining caregiving assumed to induce the implicit acquisition of somaticity and corresponding uncertainty about one’s internal states (*cf.* 2.2). Therefore, by confirming the primary role of a lack of IAw and PPC in SA and EDs, this evidence is consistent with acquiring a somatic dimension impairing interoception and increasing vulnerability to these conditions.

Hence, the above analysis corroborates H1-H2: Our evolutionarily-preordained acquisition of somaticity addresses the adaptive problem of limiting parasite threat by modulating our affiliation tendency (H1). Nonetheless, the propensity to comply and affiliate affects the vulnerability to SA and EDs. The greater this tendency, the higher the risk of developing these disorders (H2).

These hypotheses bear at least **three relevant implications** – generally valid for attachment and psychopathology but here instantiated in the case of somaticity, SAD, and EDs:

1. They bring developmental caregiving programming to attention as having a significant role in the evolution of our species and the development of psychopathology.
2. They imply a theoretical integration, adding the firmware level of analysis to the hardware (neural substrate) and software (higher cognition) ones.
3. They suggest the design of new psychological treatments.

Below, we detail each of these points.

### 5.1 Bringing developmental caregiving programming into the equation

Despite evolutionary and developmental theories being growingly detailed and comprehensive, they do not yet consider – at least operationally – caregiving as a form of biological programming resulting in acquiring implicit attachment knowledge. Our hypotheses can enhance existing theories by bringing this crucial aspect into focus.

Evolutionary principles are increasingly informing research and practice in psychiatry and clinical psychology (Liotti et al., 2017; Nesse, 2017). In particular, the phylogenetic perspective helps us classify psychological conditions by looking at the evolved mechanisms involved and their adaptive functions – i.e., their fitness enhancement. More specifically (Del Giudice and Haltigan, 2021), we can see mental disorders as the product of adaptive mechanisms designed for the species (at a population level), which can be either dysfunctional (i.e., defective in the individual) or functional (i.e., non-defective in the individual but causing a problem for another reason). When a mechanism is functional at the population level, it can be currently maladaptive and cause a problem due to an evolutionary-scale mismatch (i.e., the current context does not match the EEA). However, our hypotheses suggest a problem can also arise from a lifespan-scale mismatch.

As discussed above (*cf.* 2.2, 4.1), attachment plays a crucial role in our life history, for both early survival and later reproduction. As a durable – *firmware* – acquisition mechanism, it corresponds to the evolutionary expectation of consistency between childhood and adulthood (socio-physical) environments. However, this mechanism can become counterproductive when such an expectation is unmet. Indeed, despite attachment first acquisition being inherently adaptive, a *mismatch between early-programming and later-functioning environments* can cause a dysfunction. Given the relatively high probability of experiencing different living contexts in present times, these considerations suggest the onset of attachment-related psychopathology to be now frequently due to a **context mismatch** between the *early-programming* and *later-functioning* times. Therefore, our hypotheses shed light on how attachment can become dysfunctional and contribute to psychopathological risk over the lifespan – in particular, in relation to somaticity and SA and EDs.

### 5.2 Bridging the hardware-software gap and integrating theories

Coherently with the complexity of mental functioning and its ubiquitous impact, mental disorders are widely recognized to have a multifactorial etiology. And undoubtedly, SAD and EDs support this view (*cf.* 4.3.1, 4.3.2). Nonetheless, scientific models must adopt a particular perspective and narrow their focus to capture relevant aspects of reality. The Objectification Theory (OT) (Fredrickson and Roberts, 1997) and the Allocentric Lock Theory (ALT) (Riva, 2012) are two well-grounded and complementary theories of EDs that concentrate their attention on social – on the one hand – and neuropsychological – on the other – aspects of these conditions. We discuss here how they can be further integrated through our hypotheses H1-H2.

#### 5.2.1 Objectification theory

Objectification refers to considering a non-object as an object – namely, in regard to its physical properties. The objectified entity can be a person. And in this case, self-objectification implies representing oneself as an object, assuming a **third-person perspective**. Fredrickson and Roberts (1997) introduced OT by referring to the cultural assumptions of Western society (in general) that are informed by a woman-as-an-object conceptualization, with all the relevant consequences. According to the theory, Western culture pushes women to internalize the shared standards of female perfection – of which thinness is a crucial element – and to represent and evaluate themselves with respect to how their appearance meets such standards. As a result, women tend to self-objectify – adopting an observer’s standpoint on themselves – and be less aware of their body signals – thereby being more vulnerable to developing an ED (Fredrickson and Roberts, 1997; Calogero, 2012). Over the years, multiple studies have supported the relevance of internalizing ideal thinness and self-objectification to the development of EDs (Schaefer and Thompson, 2018).

#### 5.2.2 Allocentric lock theory

Riva (2012) proposed the ALT as a neuropsychological and neurobiological explanation for EDs, addressing the brain’s visuospatial processing related to our body representation – a key feature of eating pathologies (Serino et al., 2015). Such processing involves two reference frames – the first-person (egocentric) and third-person (allocentric) ones (*cf.* 2.1). According to the theory, the exchange of information between the working memory – holding current bodily data in an egocentric format – and the long-term memory – storing data in an allocentric format – can be impaired, favoring the latter over the former. In other words, we can suffer from an **allocentric lock**, meaning that our third-person memories can systematically override our current first-person perceptions. When this happens, memories affect perceptions, but perceptions cannot update memories. As a result, we are stuck with old representations of our body, possibly not matching its current state. The ALT suggests this impairment afflicts those suffering from EDs – who cannot update negative allocentric body memories. According to the theory, the probable origin of the lock is a dysfunction in the neural circuitry responsible for the ego-allo-centric data conversion – in particular, the Papez circuit, the retrosplenial cortex, and the parieto-occipital sulcus, which heavily rely on serotonin (Riva, 2016, 2017).

#### 5.2.3 Theoretical integration

The ALT neuropsychological perspective is entirely compatible with the OT social one. Indeed, when one retrieves visuospatial allocentric memories of themselves, they are self-objectifying by looking at themselves as a third-person would. In the case of EDs and cultural influence, the issue concerns autobiographical memories of episodes of a negative evaluation of one’s body shape compared to social standards. Thus, the ALT can explain the neuropsychological processes underpinning OT. Since somaticity concerns an uncertainty about one’s internal bodily signals that leads to referring to external figures for self-definition (*cf.* 2.2, 4.2), H1-H2 further integrate these two theories. Indeed, the somatic tends to anticipate how their reference figure wants them to be and comply with that image – thereby systematically adopting an allocentric perspective (ALT) and possibly referring to ideal thinness (OT). In other words, while tending to look at themselves from a third-person perspective by itself instantiates an allocentric lock, when the reference is ideal thinness, compliance becomes informed by such an ideal. Hence, H1-H2 provide an additional explanation – on a different level – of the phenomenology of EDs, complementing both the ALT and OT.

**Figure 3:**
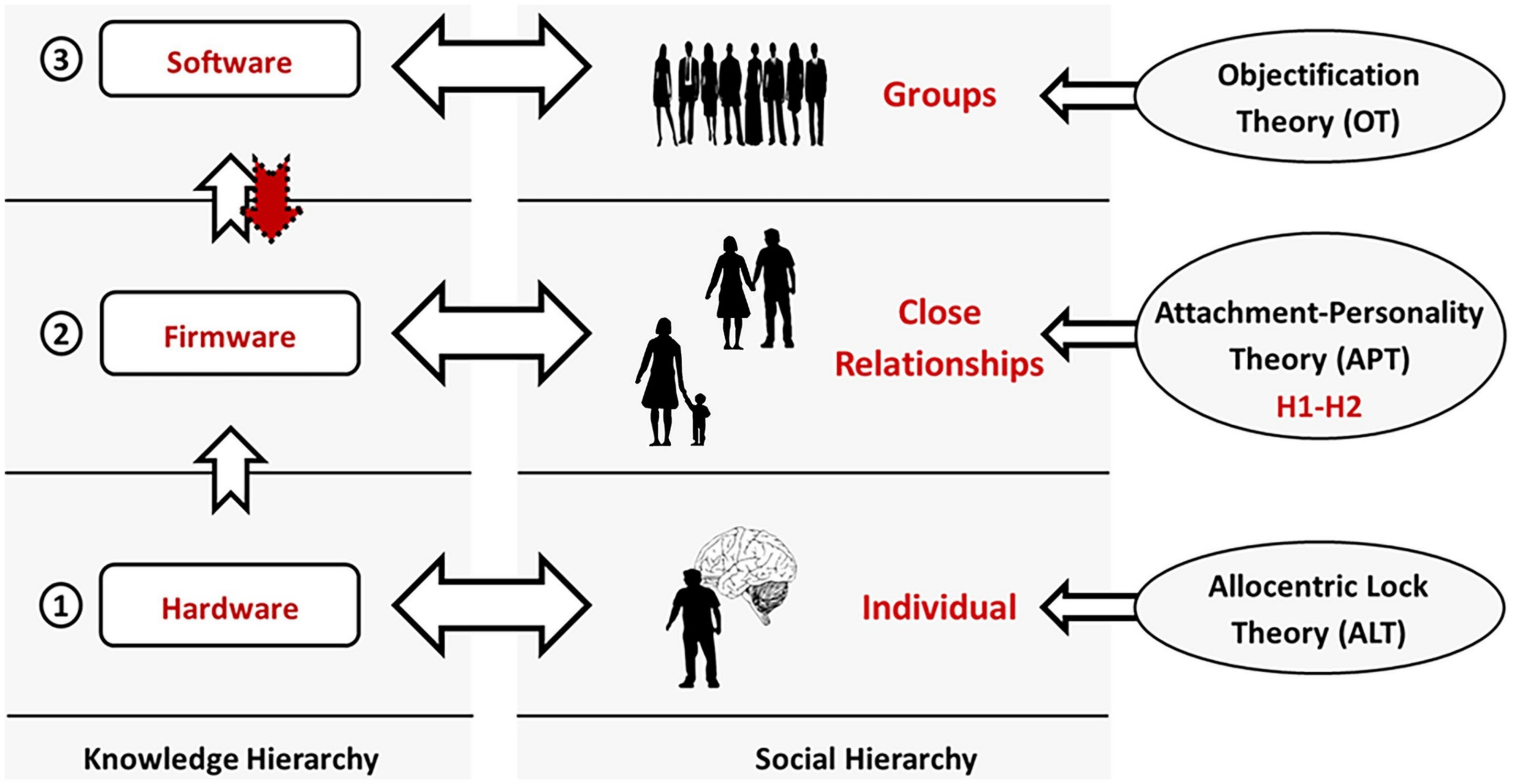
Knowledge and social hierarchies. In this work, we refer to a minimal three-layer knowledge hierarchy, distinguishing between hardware, firmware, and software (left). Each data level corresponds to a social one (center). Our neural system encodes information in its structure (hardware). Our close relationships are the source of the implicit knowledge instantiating our attachment dimensions (firmware). And groups – of any size – provide us with explicit, verbalizable information (software). Finally, each of these levels is the primary target of specific theories of mental functioning and disorders (right). H1-H2 concern the firmware level and extend the Attachment-Personality Theory (APT) with respect to somaticity. On the other hand, the Allocentric Lock Theory (ALT) and Objectification Theory (OT) focus on the hardware and software levels, respectively.

This complementarity can be further clarified through our basic and operational *brain-computer analogy* consisting of hardware, firmware, and software data levels. This hierarchical organization of knowledge corresponds to different levels of theoretical focus (Figure 3). In this regard, H1-H2 are entirely aligned with and extend the Attachment-Personality Theory (APT) (Gagliardi, 2021, 2022a) (*cf.* 2.2) with respect to somaticity. On the other hand, the two relevant etiological theories of EDs we discussed – the ALT and OT – correspond to the opposite extremes of this hierarchy – the hardware (neural substrate) and software (higher cognition) ones, respectively. H1-H2 complement them on the *firmware* (attachment) level and allow us to enhance our understanding of the multifactorial nature of EDs.

### 5.3 Enabling the design of new psychological treatments

The most relevant implication of the proposed hypotheses H1-H2 is their applicability to psychological treatments – which is allowed by the re-programmability of firmware data throughout the lifespan (*cf.* 2.2). In this regard, the clinical psychological community widely recognizes the clinical nature of attachment theory and its applicability to practice (Bowlby, 1969/1982; Gold, 2011). Consistently, many clinical schools include it in their conceptualizations and treatments. However, the theory does not provide specific indications of how it can be applied to clinical practice except for the powerful and “healing” effect of a positive attachment relationship – often called a “*corrective emotional experience*” (Berry and Danquah, 2016). By relying on the concepts of sensitive periods and imprinting, we can provide further specifications on how attachment can be applied in therapy.

#### 5.3.1 Imprinting conditions

The implicit information constituting the attachment dimensions is first acquired – during attacher-caregiver interactions – in evolutionarily preordained, early sensitive periods (*cf.* 2.2). The firmware nature of the acquisition process entails meeting the following conditions: (1) The attachment motivational system must be active. (2) A dimension-related situation must be experienced. (3) Such a situation must be perceived as adaptation-relevant. Imprinting is an evolutionary, life-saving, domain-specific learning mechanism. In the case of avoidance and ambivalence, the attachment-caregiving interaction concerns the provision of emotional care and physical availability, respectively (*cf.* 4.1). On the other hand, somaticity regards self-definition, which involves the implicit and explicit elements of the interaction related to the acknowledgment of self-expression.

#### 5.3.2 Later imprinting

Even if it is harder to realize, a later imprinting is possible (*cf.* 2.2). Indeed, although the parent–child relationship is foundational, similar relational contexts can elicit a change – for instance, a romantic or psychotherapeutic relationship (corrective emotional experience). Therefore, to rewrite the dimensions after a sensitive period, we expect the same conditions as in the first imprinting to be necessary. Using a metaphor, the neurocircuits that once were relatively easy to carve become harder to shape but still need the same chisel. In the case of individual therapy, treatment will need to follow the steps listed above to allow for the correction of the patient’s emotional experience. The therapist will need to: (1) Build a relationship – based on safety, authenticity, and trust – that works as an attachment relationship. (2) Identify and selectively elicit the dimension requiring a change in the relationship. (3) Act as a positive attachment figure in an adaptation-relevant situation.

As discussed above, a dimension will be imprinted when the circumstances are perceived as critical (and unexpected) with respect to such a dimension (i.e., adaptation-relevant). Regarding somaticity, this often concerns psychological control and inhibition of self-expression (*cf.* 4.3). For example, if a child genuinely expresses an emotion – say, happiness – and the parent ignores it and even overrides it with another – disappointment, for instance – then the child will probably perceive the situation as highly disorienting in terms of their internal states. In this case, this event could elicit the imprinting of somaticity. Given the possible somatic origin of SAD and EDs, the imprinting conditions for somaticity can be the target of an attachment-oriented treatment for these disorders. In this respect, a powerful technique that seems particularly adequate to reprogram attachment dimensions – and somaticity, in particular – is **imagery rescripting**, consisting of evoking in one’s mind an unresolved problematic autobiographical event and rescripting it by substituting the original helplessness experience with a new empowered one (Young et al., 2003; Arntz, 2012). The technique has been proven effective in reducing symptoms in a wide range of disorders, including SAD and EDs (Morina et al., 2017; Lloyd and Marczak, 2022).

### 5.4 Limitations and future work

This work integrates evidence from multiple fields to formulate two developmental-evolutionary hypotheses, H1-H2. As such, it is primarily limited by being a purely theoretical effort, although our evaluation of H1-H2 suggests various ways of testing them. Concerning H1, no studies yet addressed the connection between caregiving/somaticity and larger-scale social phenomena. This gap was partially filled by the literature we examined (e.g., PST and family ties). But specific statistical analyses will allow us to shed further light on the strength of relevant relationships, such as between somaticity and the degree of specific group affiliations. Experimental psychology can also help investigate this hypothesis by, for example, looking at the effect of psychological control on our affiliation tendency. Concerning H2, a significant limitation is the multifactorial origin of psychological disorders, which complicates isolating its causes. In this case, however, clinical tools can be designed to investigate the connections between childhood caregiving experiences and current vulnerability to specific mental conditions. In this regard, we have developed such an inventory (Gagliardi, 2022a) and are now proceeding with a large-scale administration. An additional testing tool is computational modeling, through which the effects of attachment-related assumptions on behavior can be tested against available data (Gagliardi, 2022b). Despite being beyond the scope of this paper, its indispensable follow-up must be extended testing on multiple fronts.

## 6 Conclusion

In this work, we propose and discuss two hypotheses (H1-H2) on the origin and implications of the human tendency to comply and affiliate. A growing body of research leads us to suggest the following. Human beings are programmable biological machines whose primary programming occurs in the context of attachment relationships. Its dimensions were designed by evolution according to specific selective pressures. Rather than a monolithic block aimed at maintaining proximity, attachment is a motivational and data system infants and children use to collect information from their caregivers on several domains, optimize the relationship, and orient their future life. Each dimension concerns a specific adaptive goal and the modulation of a related motivational system. Somaticity is about modulating affiliation as an ancient tool to prevent parasitic threats. Nonetheless, a mismatch between the current and learning contexts can result in an excessive tendency to seek external references and enhanced vulnerability to social anxiety and eating disorders. Taking this adaptive feature into explicit account entails relevant theoretical and methodological implications for evolutionary psychiatry and clinical psychology. In particular, H1-H2 are consistent and complementarity to the well-grounded *objectification* and *allocentric lock* theories and applicable to the design of new psychological treatments. Without aiming to prove them, our analysis of multidisciplinary evidence supports the presented hypotheses and urges their direct testing.

## Data availability statement

The original contributions presented in the study are included in the article/supplementary material, further inquiries can be directed to the corresponding author.

## Author contributions

MG: Writing – original draft, Writing – review & editing.

## Footnotes

^1^One’s impairment in identifying and describing their own emotions and tendency to orient their thinking externally.
